# Impact of hospital-acquired acute kidney injury on Covid-19 outcomes in patients with and without chronic kidney disease: a multicenter retrospective cohort study

**DOI:** 10.3906/sag-2011-169

**Published:** 2021-06-28

**Authors:** Savaş ÖZTÜRK, Kenan TURGUTALP, Mustafa ARICI, Hakkı ÇETİNKAYA, Mehmet Rıza ALTIPARMAK, Zeki AYDIN, Zeki SOYPAÇACI, Feyza BORA, Ekrem KARA, Egemen CEBECİ, Tuba Elif ÖZLER, Mürşide Esra DÖLARSLAN, Savaş SİPAHİ, Yavuz AYAR, İdris ŞAHİN, Serkan BAKIRDÖĞEN, Mahmud İSLAM, Numan GÖRGÜLÜ, Melike Betül ÖĞÜTMEN, Erkan ŞENGÜL, Özkan GÜNGÖR, Nurhan SEYAHİ, Bülent TOKGÖZ, Ali Rıza ODABAŞ, Halil Zeki TONBUL, Siren SEZER, Alaattin YILDIZ, Kenan ATEŞ

**Affiliations:** 1 Department of Nephrology, University of Health Sciences, Haseki Training and Research Hospital, İstanbul Turkey; 2 Division of Nephrology, Department of Internal Medicine, Mersin University Faculty of Medicine, Training and Research Hospital, Mersin Turkey; 3 Department of Nephrology, Faculty of Medicine, Hacettepe University, Ankara Turkey; 4 Department of Nephrology, Sultan 2. Abdulhamid Han Training and Research Hospital, University of Health Sciences, İstanbul Turkey; 5 Division of Nephrology, Department of Internal Medicine, Cerrahpaşa Faculty of Medicine, İstanbul University–Cerrahpaşa, İstanbul Turkey; 6 Department of Nephrology, Darıca Farabi Training and Research Hospital, Kocaeli Turkey; 7 Division of Nephrology, Department of Internal Medicine, İzmir Katip Çelebi University, Atatürk Training and Research Hospital, İzmir Turkey; 8 Division of Nephrology, Department of Internal Medicine, Faculty of Medicine, Akdeniz University, Antalya Turkey; 9 Division of Nephrology, Department of Internal Medicine, Faculty of Medicine, Recep Tayyip Erdoğan University, Rize Turkey; 10 Division of Nephrology, Department of Internal Medicine, Kanuni Sultan Süleyman Training and Research Hospital, University of Health Sciences, İstanbul Turkey; 11 Department of Nephrology, Trabzon Kanuni Training and Research Hospital, University of Health Sciences, Trabzon Turkey; 12 Division of Nephrology, Department of Internal Medicine, Sakarya University Training and Research Hospital, Sakarya Turkey; 13 Department of Nephrology, Bursa City Hospital, Bursa Turkey; 14 Division of Nephrology, Department of Internal Medicine, Turgut Özal Medical Center, İnönü University Faculty of Medicine, Malatya Turkey; 15 Division of Nephrology, Department of Internal Medicine, Çanakkale Onsekiz Mart University, Çanakkale Turkey; 16 Department of Nephrology, Zonguldak Atatürk State Hospital, Zonguldak Turkey; 17 Division of Nephrology, Department of Internal Medicine, Bağcılar Training and Research Hospital, University of Health Sciences, İstanbul Turkey; 18 Department of Nephrology, Haydarpaşa Numune Training and Research Hospital, University of Health Sciences, İstanbul Turkey; 19 Department of Nephrology, Kocaeli Derince Training and Research Hospital, University of Health Sciences, Kocaeli Turkey; 20 Department of Nephrology, Faculty of Medicine, Kahramanmaraş Sütçü İmam University, Kahramanmaraş Turkey; 21 Department of Nephrology, Faculty of Medicine, Erciyes University, Kayseri Turkey; 22 Department of Nephrology, Meram Faculty of Medicine, Necmettin Erbakan University, Konya Turkey; 23 Division of Nephrology, Department of Internal Medicine, Faculty of Medicine, Atılım University, Ankara Turkey; 24 Division of Nephrology, Department of Internal Medicine, İstanbul Faculty of Medicine, İstanbul University, İstanbul Turkey; 25 Division of Nephrology, Department of Internal Medicine, Faculty of Medicine, Ankara University, Ankara Turkey

**Keywords:** Acute kidney injury, chronic kidney disease, Covid-19, hospitalization, mortality

## Abstract

**Background/aim:**

Hospital-acquired acute kidney injury (HA-AKI) may commonly develop in Covid-19 patients and is expected to have higher mortality. There is little comparative data investigating the effect of HA-AKI on mortality of chronic kidney disease (CKD) patients and a control group of general population suffering from Covid-19.

**Materials and methods:**

HA-AKI development was assessed in a group of stage 3–5 CKD patients and control group without CKD among adult patients hospitalized for Covid-19. The role of AKI development on the outcome (in-hospital mortality and admission to the intensive care unit [ICU]) of patients with and without CKD was compared.

**Results:**

Among 621 hospitalized patients (age 60 [IQR: 47–73]), women: 44.1%), AKI developed in 32.5% of the patients, as stage 1 in 84.2%, stage 2 in 8.4%, and stage 3 in 7.4%. AKI developed in 48.0 % of CKD patients, whereas it developed in 17.6% of patients without CKD. CKD patients with HA-AKI had the highest mortality rate of 41.1% compared to 14.3% of patients with HA-AKI but no CKD (p < 0.001). However, patients with AKI+non-CKD had similar rates of ICU admission, mechanical ventilation, and death rate to patients with CKD without AKI. Adjusted mortality risks of the AKI+non-CKD group (HR: 9.0, 95% CI: 1.9–44.2) and AKI+CKD group (HR: 7.9, 95% CI: 1.9–33.3) were significantly higher than that of the non-AKI+non-CKD group.

**Conclusion:**

AKI frequently develops in hospitalized patients due to Covid-19 and is associated with high mortality. HA-AKI has worse outcomes whether it develops in patients with or without CKD, but the worst outcome was seen in AKI+CKD patients.

## 1. Introduction

Acute kidney injury (AKI) may commonly develop during the course of Covid-19, specifically in severe cases. There is great heterogeneity in papers reporting hospital-acquired acute kidney injury (HA-AKI) prevalence and mortality rates. In earlier studies, mainly from China, the pooled incidence of AKI was relatively lower, being reported in 3% of hospitalized patients and 19% of patients admitted to the intensive care unit (ICU) [1]. In a study including 701 patients from Wuhan, AKI developed in 5.1% of the patients during hospitalization and was associated with an increased risk of in-hospital mortality [2]. In the same study, as the AKI stage increased, adjusted in-hospital mortality risk was also increased. In another study from Wuhan, AKI developed in 15% of the patients, with a rate of 50% in nonsurvivors but only 1% of the survivors [3]. A recent study from New York showed a higher incidence of AKI; AKI incidence was found in 36.6% of 5449 hospitalized patients with Covid-19, most of the AKI (46.5%) cases were stage I [4]. 

Chronic kidney disease (CKD) is one of the most important risk factors for the development of HA-AKI. There is, however, insufficient data for the development of AKI on CKD during Covid-19. Earlier studies from China had few CKD cases. In the recent New York study with the highest AKI incidence, CKD patients were not included as comorbidity due to the diagnostic difficulties. On the other hand, the incidence of AKI, typical clinical, laboratory, and characteristic findings in Covid-19 patients hospitalized inside or outside of ICU is still an area of uncertainty [5]. We, therefore, aimed to investigate the development of AKI during hospitalization and its effect on survival in hospitalized patients with Covid-19 in a cohort that included patients with known CKD compared with a control group comprising patients without CKD.

## 2. Materials and methods

### 2.1. Design and participants

This study included consecutive hospitalized patients selected among adult Covid-19 patients who were recorded in an ongoing national multicenter retrospective database collected from 47 centers. The study was unconditionally supported by the Turkish Society Nephrology. Stage 3–5 CKD patients, hemodialysis, peritoneal dialysis, renal transplantation, and control group patients without kidney disease were registered to the database. This study has included only the stage 3–5 CKD patients and patients without CKD. Patients who had AKI on admission, younger than 18 years of age, lack hospital discharge information or survival data, being still hospitalized (except intensive care unit [ICU]) at the time of data collection and pregnant patients were excluded from the study. Patients with insufficient data to diagnose AKI during hospitalization were also excluded. All patients in this database were assessed, diagnosed, followed up, and recorded to the database by the attending nephrologists of the participating centers. Local ethics committee approval was obtained for the study. 

### 2.2. Data collection and definitions

All data were collected by reviewing electronic health records of participating centers’ hospital systems. The data including demographics, symptoms on admission, comorbidities and medications, smoking habits, primary kidney disease of the CKD patients, initial laboratory tests, including serum creatinine, estimated glomerular filtration rate (GFR) calculated with the CKD-EPI formula [6], serum albumin, ferritin, C-reactive protein (CRP), hemoglobin, lymphocyte, and platelet counts were gathered. Data regarding the clinical severity of Covid-19 at the presentation, which was categorized according to our national guideline [7], drug treatments given for Covid-19, and outcomes (ICU admission, mechanical ventilation, discharge, or death) were also collected. 

Kidney Disease: Improving Global Outcomes (KDIGO) criteria were used for stage 3–5 CKD and the AKI definitions [8,9]. KDIGO defines patients with low GFR (<GFR <60 mL/ min/1.73 m2) for more than 3 months as CKD regardless of the kidney damage markers. The diagnosis of CKD was accepted according to the investigators’ statement, no obligation was imposed on diagnostic tools and markers. Urine criteria were not used for the diagnosis of AKI as following urine output was not possible during the pandemic period for most patients. 

The patients were diagnosed as Covid-19, according to “National Covid-19 Diagnosis and Treatment Guideline” [7]. In this guide, briefly, patients who have positive nasopharyngeal swap SARS-CoV-2 PCR test are accepted as “confirmed cases”. In patients whose PCR tests are negative, if chest computed tomography is found to be compatible with the viral infection along with the clinical findings, patients are accepted as “probable cases” and managed as confirmed cases. We have included all confirmed and probable cases to the study.

### 2.3. Statistical analysis

The analyses were performed using the IBM SPSS Statistics for Windows, Version 25.0 (IBM Corp., Armonk, NY, USA). Descriptive statistics were expressed as numbers and percentages for categorical variables and as mean, standard deviation, median, minimum-maximum, and 25th percentile-75th percentile for numerical variables. The conformity of variables to normal distribution was assessed using visual (histogram and probability graphs) and analytical methods (Kolmogorov–Smirnov/Shapiro–Wilk tests). For multiple group comparisons of categorical variables, the chi-square test was used when chi-square condition was met and Fisher’s exact test was used when the chi-square condition was not met. In multiple group comparisons of numerical variables, the analysis of variance (ANOVA) test was used for normally distributed numerical variables and the Kruskal–Wallis test was used for nonnormally distributed numerical variables. In post-hoc analysis, the Mann–Whitney U test with Bonferroni correction was used for subgroup analysis of nonnormally distributed variables, and the chi-square test with Bonferroni correction was used for subgroup analysis of categorical variables. Survival analysis was performed using the Kaplan–Meier method. Log-rank test was used for the comparison of the survival durations among the AKI-CKD groups. Multivariate Cox regression analysis was performed using the clinical parameters to be related to survival in the univariate analysis. Final multivariate models were derived using stepwise backward LR method from the initial model created with the candidate variables in Cox regression analysis. Logistic regression analysis was used with the enter method to determine risk factors associated with the presence of AKI. The statistical significance level was set at p < 0.05.

## 3. Results

### 3.1. Demographic and clinical characteristics

A total of 741 hospitalized patients were registered to the database as CKD or control groups. Among these, 106 patients with incomplete data for the diagnosis of AKI and 14 patients with various reasons (3 patients due to lack of discharge information or survival data, 10 patients due to AKI on admission, 1 patient due to pregnancy) were excluded from the study. The remaining 621 patients (median age: 60 [IQR: 47–73]), women: 44.1%) were included in the analyses.

### 3.2. AKI development 

Hospital-acquired AKI developed in 202 (32.5%) of the patients. The AKI stages were as follows: stage 1 in 170/202 (84.2%), stage 2 in 17/202 (8.4%), and stage 3 in 15/202 (7.4%). Table 1 shows the baseline demographic and clinical characteristics and laboratory values of the patients with and without AKI. Patients who developed HA-AKI were older, and their comorbidities (diabetes mellitus, hypertension, cardiac diseases) were higher than the patients without AKI. Antihypertensive and antidiabetic medication usages were higher in the AKI group due to the higher hypertension and DM rate in this group. Their baseline serum creatinine, CRP, and ferritin levels were higher, serum albumin and hemoglobin levels and lymphocyte count were lower in AKI patients. The baseline eGFR was significantly lower in the patients developing AKI during hospitalization, i.e. the lower the eGFR degree in baseline evaluation, the higher the incidence of AKI during hospitalization. There were a total of 304 CKD patients, and HA-AKI developed in 48.0% (146) of these patients, whereas it developed only in 17.6% (56) of 317 patients without CKD.

**Table 1:** Baseline demographic, clinical characteristics and laboratory values of the patients according to the presence of AKI.

Characteristic		All Patients(N = 621)	No AKI(N = 419)	AKI(N = 202)	p
Demographic information					
Age (years), median (Q1-Q3)		60 (47–73)	58 (44–71)	65 (53–75)	<0.001
Sex, n/N (%)	Female	274/621 (44.1)	194/419 (46.3)	80/202 (39.6)	0.115
Smoking, n/N (%)	Never smoking	391/455 (85.9)	271/306 (88.6)	120/149 (80.5)	0.021
	Current smoker	64/455 (14.1)	35/306 (11.4)	29/149 (19.5)	
Coexisting disorder, n/N (%)	Diabetes mellitus	172/614 (28.0)	96/417 (23.0)	76/197 (38.6)	<0.001
	Hypertension	332/615 (54.0)	186/415 (44.8)	146/200 (73.0)	<0.001
	Ischemic heart disease	139/598 (23.2)	66/409 (16.1)	73/189 (38.6)	<0.001
	Heart failure	78/603 (12.9)	33/411 (8.0)	45/192 (23.4)	<0.001
	Ischemic heart disease/heart failure	161/602 (26.7)	73/409 (17.8)	88/193 (45.6)	<0.001
	COPD	83/606 (13.7)	51/415 (12.3)	32/191 (16.8)	0.137
	Cancer	32/602 (5.3)	20/413 (4.8)	12/189 (6.3)	0.444
	Chronic liver disease	5/609 (0.8)	3/415 (0.7)	2/194 (1.0)	0.656
Medications, n/N (%)	ACE inhibitors	103/572 (18.0)	62/396 (15.7)	41/176 (23.3)	0.028
	ARBs	106/569 (18.6)	59/391 (15.1)	47/178 (26.4)	<0.001
	Calcium channel blockers	186/580 (32.1)	99/399 (24.8)	87/181 (48.1)	<0.001
	Beta-blockers	153/578 (26.5)	78/394 (19.8)	75/184 (40.8)	<0.001
	Other ihypertensives	85/560 (15.2)	53/391 (13.6)	32/169 (18.9)	0.103
	Insulin	83/571 (14.5)	41/397 (10.3)	42/174 (24.1)	<0.001
	Oral antidiabetics	98/580 (16.9)	63/401 (15.7)	35/179 (19.6)	0.254
	Statins	86/562 (15.3)	41/390 (10.5)	45/172 (26.2)	<0.001
	Antiaggregant or anticoagulants	188/579 (32.5)	108/398 (27.1)	80/181 (44.2)	<0.001
Primary kidney disease, n/N (%)	Diabetic nephropathy	83/217 (38.2)	41/107 (38.3)	42/110 (38.2)	0.213
	Hypertensive ephrosclerosis	110/217 (50.7)	56/107 (52.3)	54/110 (49.1)	
	ADPKD	2/217 (0.9)	2/107 (1.9)	0/110 (0.0)	
	Primary glomerular disease	10/217 (4.6)	2/107 (1.9)	8/110 (7.3)	
	Urologic diseases	8/217 (3.7)	3/107 (2.8)	5/110 (4.5)	
	Other	4/217 (1.8)	3/107 (2.8)	1/110 (0.9)	
Laboratory findings, Median (Q1-Q3)	Creatinine (µmol/L)	90.17 (70.72–141.44)	79.56 (66.30–114.92)	133.93 (88.40–189.18)	<0.001
	Albumin (g/L)	37.0 (33.0–40.0)	38.0 (35.0–41.0)	35.0 (30.0–39.5)	<0.001
	Ferritin (µg/L)	230.00 (102.0–496.0)	195.5 (87.0–436.8)	320.90 (140.0–628.3)	<0.001
	Haemoglobin (g/L)	123.2 ± 21.1	126.5 ± 20.2	116.4 ± 21.2	<0.001
	Lymphocyte count (×109/L)	1.200 (0.75–1.770)	1.250 (0.810–1.800)	1.090 (0.640–1.650)	0.007
	Platelet count (×109/L)	223 (176–276)	221 (176–266)	232 (175–300)	0.100
C-reactive protein, n/N (%)	<10	411/621 (66.2)	307/419 (73.3)	104/202 (51.5)	<0.001
(× upper limit)	≥10	210/621 (33.8)	112/419 (26.7)	98/202 (48.5)	
Baseline eGFR (mL/min/1.73m2), n / N (%)	<15	117/621 (18.8)	53/419 (12.6)	64/202 (31.7)	<0.001
	15–30	74/621 (11.9)	38/419 (9.1)	36/202 (17.8)	
	30–60	113/621 (18.2)	67/419 (16.0)	46/202 (22.8)	
	60–90	102/621 (16.4)	80/419 (19.1)	22/202 (10.9)	
	>90	215/621 (34.6)	181/419 (43.2)	34/202 (16.8)	
Time between first symptom and diagnosis (days), median (Q1–Q3)		3 (2–5)	3 (2–5)	3 (2–5)	0.482
Clinical presentation, n / N (%)	Mild-moderate disease	435/619 (70.3)	337/418 (80.6)	98/201 (48.8)	<0.001
	Severe-critical disease	184/619 (29.7)	81/418 (19.4)	103/201 (51.2)	
Radiological confirmation n/N (%)		530/621 (85.3)	354/419 (84.5)	176/202 (87.1)	0.383
PCR confirmation, n/N (%)		382/621 (61.5)	245/419 (58.5)	137/202 (67.8)	0.025
Drug treatments, n/N (%)	Oseltamivir	423/568 (74.5)	280/395 (70.9)	143/173 (82.7)	0.003
	Macrolides	543/597 (91)	373/405 (92.1)	170/192 (88.5)	0.157
	Hydroxychloroquine	608/619 (98.2)	412/418 (98.6)	196/201 (97.5)	0.348
	Lopinavir-ritonavir	31/456 (6.8)	9/314 (2.9)	22/142 (15.5)	<0.001
	Favipiravir	201/520 (38.7)	94/342 (27.5)	107/178 (60.1)	<0.001
	Glucocorticoids	35/450 (7.8)	8/312 (2.6)	27/138 (19.6)	<0.001
	Tocilizumab	12/448 (2.7)	3/313 (1.0)	9/135 (6.7)	0.002
	Convalescent plasma	1/442 (0.2)	0/311 (0.0)	1/131 (0.8)	-
	Canakinumab/anakinra	1/444 (0.2)	0/311 (0.0)	1/133 (0.8)	-
Laboratory tests during hospitalization, n / N (%)	Leukopenia <4.00/×109/L)	100/615 (16.3)	65/415 (15.7)	35/200 (17.5)	0.563
	Lymphopenia (<1.5/×109/L)	329/613 (53.7)	191/412 (46.4)	138/201 (68.7)	<0.001
	Anemia (hemoglobin < 100 g/L)	166/615 (27.0)	67/414 (16.2)	99/201 (49.3)	<0.001
	Thrombocytopenia (<150.00×109/L)	96/614 (15.6)	45/414 (10.9)	51/200 (25.5)	<0.001
	LDH (>2×upper limit of normal)	158/603 (26.2)	62/408 (15.2)	96/195 (49.2)	<0.001
	AST (>2×upper limit of normal)	129/612 (21.1)	55/412 (13.3)	74/200 (37.0)	<0.001
Highest value of CRP level during follow-up, n / N (%)	Normal	96/619 (15.5)	78/418 (18.7)	18/201 (9.0)	<0.001
(x of upper normal value)	1–5	151/619 (24.4)	117/418 (28)	34/201 (16.9)	
	5–10	85/619 (13.7)	63/418 (15.1)	22/201 (10.9)	
	10–20	120/619 (19.4)	83/418 (19.9)	37/201 (18.4)	
	> 20	167/619 (27.0)	77/418 (18.4)	90/201 (44.8)	
Length of stay at hospital, Median (Q1–Q3)		9 (6–14)	8 (6–11)	12 (8–16)	<0.001
ICU admission, n / N (%)		125/621 (20.1)	40/419 (9.5)	85/202 (42.1)	<0.001
Mechanical ventilation, n / N (%)		95/124 (76.6)	22/40 (55.0)	73/84 (86.9)	<0.001
Outcome, n / N (%)	Dead	86/621 (13.8)	18/419 (4.3)	68/202 (33.7)	<0.001
	Discharged	518/621 (83.4)	393/419 (93.8)	125/202 (61.9)	
	Transfer to another center	7/621 (1.1)	4/419 (1.0)	3/202 (1.5)	
	Still in ICU	10/621 (1.6)	4/419 (1.0)	6/202 (3.0)	

### 3.3. Survival data

A total of 86 (13.8%) patients died, 518 (83.4%) recovered, 7 (1.1%) were transferred to another center, and 10 (1.6%) were still in ICU at the time data was collected. ICU admission rate, need for mechanical ventilation, and in-hospital mortality were significantly higher in AKI-developing patients (85/125 [68.0%], 73/84 [76.8%], and 68/86 [79.1%], respectively). When the patients who died or discharged were compared, the same parameters that were related to AKI development were almost found to be associated with mortality (Table 2).

**Table 2:** Characteristics and laboratory values of patients according to survival.

Characteristic		All patients(N = 604)	Recovered(N = 518)	Died(N = 86)	p
Demographic information					
Age (years), median (Q1–Q3)		60 (47–72)	58 (46–70)	73 (63–80)	<0.001
Sex, n/N (%)	Female	267/604 (44.2)	238/518 (45.9)	29/86 (33.7)	0.035
Smoking, n/N (%	Not smoking	386/447 (86.4)	337/394 (85.5)	49/53 (92.5)	0.168
	Smoking	61/447 (13.6)	57/394 (14.5)	4/53 (7.5)	
Coexisting disorder, n/N (%)	Diabetes mellitus	166/598 (27.8)	129/514 (25.1)	37/84 (44.0)	<0.001
	Hypertension	319/598 (53.3)	249/513 (48.5)	70/85 (82.4)	<0.001
	Ischemic heart disease	132/583 (22.6)	92/505 (18.2)	40/78 (51.3)	<0.001
	Heart failure	74/588 (12.6)	48/508 (9.4)	26/80 (32.5)	<0.001
	Ischemic heart disease/heart failure	153/587 (26.1)	108/507 (21.3)	45/80 (56.3)	<0.001
	COPD	79/592 (13.3)	63/512 (12.3)	16/80 (20.0)	0.060
	Cancer	31/588 (5.3)	25/509 (4.9)	6/79 (7.6)	0.288
	Chronic liver disease	5/594 (0.8)	3/512 (0.6)	2/82 (2.4)	0.143
Primary kidney disease, n/N (%)	Diabetic nephropathy	82/211 (38.9)	57/148 (38.5)	25/63 (39.7)	0.430
	Hypertensive nephrosclerosis	105/211 (49.8)	70/148 (47.3)	35/63 (55.6)	
	ADPKD	2/211 (0.9)	2/148 (1.4)	0/63 (0.0)	
	Primary glomerular disease	10/211 (4.7)	9/148 (6.1)	1/63 (1.6)	
	Urologic diseases	8/211 (3.8)	6/148 (4.1)	2/63 (3.2)	
	Other	4/211 (1.9)	4/148 (2.7)	0/63 (0.0)	
Medications, n/N (%)	ACE inhibitors	96/556 (17.3)	78/487 (16.0)	18/69 (26.1)	0.038
	ARBs	101/552 (18.3)	84/485 (17.3)	17/67 (25.4)	0.110
	Calcium channel blockers	178/563 (31.6)	140/491 (28.5)	38/72 (52.8)	<0.001
	Beta-blockers	146/562 (26.0)	111/488 (22.7)	35/74 (47.3)	<0.001
	Other antihypertensives	82/543 (15.1)	61/478 (12.8)	21/65 (32.3)	<0.001
	Insulin	79/556 (14.2)	62/488 (12.7)	17/68 (25.0)	0.007
	Oral antidiabetics	94/564 (16.7)	76/492 (15.4)	18/72 (25.0)	0.042
	Statins	80/547 (14.6)	66/481 (13.7)	14/66 (21.2)	0.106
	Antiaggregant or anticoagulants	180/563 (32.0)	139/486 (28.6)	41/77 (53.2)	<0.001
Laboratory findings, median (Q1–Q3)	Creatinine (µmol/L)	89.28 (70.28–141.44)	83.10 (67.18–129.06)	102.99 (109.62 – 212.16)	<0.001
	Albumin (g/L)	37.0 (33.0–40.0)	38.0 (34.0–41.0)	32.0 (27.0–36.0)	<0.001
	Ferritin (µg/L)	230.0 (99.0–498.0)	199.9 (92.5–451.0)	450.0 (250.0–1127.5)	<0.001
	Hemoglobin (g/L)	123.4 ± 20.9	124.7 ± 20.7	115.7 ± 20.9	<0.001
	Lymphocyte count (x109/L)	1.200 (0.750–1.770)	1.245 (0.830–1.800)	0.750 (0.480–1.310)	<0.001
	Platelet count (x109/L)	224 (176–276)	226 (178–275)	208 (157–279)	0.148
eGFR (mL/min/1.73 m2), median (Q1–Q3)	<15	111/604 (18.4)	81/518 (15.6)	30/86 (34.9)	<0.001
	15–30	69/604 (11.4)	48/518 (9.3)	21/86 (24.4)	
	30–60	112/604 (18.5)	87/518 (16.8)	25/86 (29.1)	
	60–90	101/604 (16.7)	96/518 (18.5)	5/86 (5.8)	
	>90	211/604 (34.9)	206/518 (39.8)	5/86 (5.8)	
C-reactive protein, n/N (%)	Normal	117/604 (19.4)	115/518 (22.2)	2/86 (2.3)	<0.001
(× upper limit)	1–5	190/604 (31.5)	182/518 (35.1)	8/86 (9.3)	
	5–10	95/604 (15.7)	82/518 (15.8)	13/86 (15.1)	
	10–20	106/604 (17.5)	82/518 (15.8)	24/86 (27.9)	
	>20	96/604 (15.9)	57/518 (11.0)	39/86 (45.3)	
Time between first symptom and diagnosis (days), median (Q1–Q3)		3 (2–5)	3 (2–5)	3.5 (2.5–6)	0.327
Clinical presentation, n / N (%)	Mild-moderate disease	427/602 (70.9)	418/517 (80.9)	9/85 (10.6)	<0.001
	Severe-critical disease	175/602 (29.1)	99/517 (19.1)	76/85 (89.4)	
Radiological confirmation n/N (%)		515/604 (85.3)	435/518 (84.0)	80/86 (93)	0.028
PCR confirmation, n/N (%)		371/604 (61.4)	313/518 (60.4)	58/86 (67.4)	0.216
Drug treatments, n/N (%)	Oseltamivir	412/552 (74.6)	341/471 (72.4)	71/81 (87.7)	0.004
	Macrolides	530/580 (91.4)	453/500 (90.6)	77/80 (96.3)	0.095
	Hydroxychloroquine	591/602 (98.2)	509/517 (98.5)	82/85 (96.5)	0.194
	Lopinavir-ritonavir	31/443 (7.0)	12/381 (3.1)	19/62 (30.6)	<0.001
	Favipiravir	191/504 (37.9)	128/428 (29.9)	63/76 (82.9)	<0.001
	Glucocorticoids	33/438 (7.5)	11/382 (2.9)	22/56 (39.3)	<0.001
	Tocilizumab	12/435 (2.8)	4/381 (1.0)	8/54 (14.8)	<0.001
	Convalescent plasma	1/430 (0.2)	0/379 (0.0)	1/51 (2.0)	-
	Canakinumab/anakinra	1/432 (0.2)	0/380 (0.0)	1/52 (1.9)	-
Laboratory tests during hospitalization, n / N (%)	Leukopenia (<4.00/×109/L)	96/598 (16.1)	77/512 (15.0)	19/86 (22.1)	0.099
	Lymphopenia (<1.5/×109/L)	318/596 (53.4)	241/511 (47.2)	77/85 (90.6)	<0.001
	Anemia (hemoglobin < 100 g/L)	156/598 (26.1)	95/513 (18.5)	61/85 (71.8)	<0.001
	Thrombocytopenia (<150.00×109/L)	94/597 (15.7)	54/511 (10.6)	40/86 (46.5)	<0.001
	LDH (>2×upper limit of normal)	150/586 (25.6)	89/503 (17.7)	61/83 (73.5)	<0.001
	AST (>2×upper limit of normal)	126/595 (21.2)	76/509 (14.9)	50/86 (58.1)	<0.001
Highest value of CRP level during follow-up, n / N (%)	Normal	95/602 (15.8)	94/516 (18.2)	1/86 (1.2)	<0.001
(x of upper normal value)	1–5	148/602 (24.6)	145/516 (28.1)	3/86 (3.5)	
	5–10	85/602 (14.1)	82/516 (15.9)	3/86 (3.5)	
	10–20	118/602 (19.6)	104/516 (20.2)	14/86 (16.3)	
	>20	156/602 (25.9)	91/516 (17.6)	65/86 (75.6)	
AKI development, n/N (%)	No AKI	411/604 (68.0)	393/518 (75.9)	18/86 (20.9)	<0.001
	AKI (any stage)	193/604 (32.0)	125/518 (24.1)	68/86 (79.1)	<0.001
	Stage 1	162/604 (26.8)	116/518 (22.4)	46/86 (53.5)	
	Stage 2	16/604 (2.6)	5/518 (1.0)	11/86 (12.8)	
	Stage 3	15/604 (2.5)	4/518 (0.8)	11/86 (12.8)	
ICU admission, n / N (%)		114/604 (18.9)	32/518 (6.2)	82/86 (95.3)	<0.001
Mechanical ventilation, n / N (%)		87/114 (76.3)	6/32 (18.8)	81/82 (98.8)	<0.001

When patients were grouped according to the presence or absence of AKI, the AKI+CKD group consisted of patients who had worse clinical and laboratory parameters at the admission, and they had worse outcomes (Table 3). The outcomes of ICU admission, mechanical ventilation, and death rate of the patients with AKI but no CKD, was almost similar and not statistically different than patients with CKD and no AKI (11/56 [19.6%] vs. 28/158 [17.7%]; 8/10 [80%] vs. 19/28 [67.9%]; and 8/56 [14.3%] vs.16/158 [10.1%], respectively) (Table 3). The non-AKI+non-CKD group had better laboratory and clinical parameters and showed better outcomes. Only 2 of 261 (0.8%) patients died in this group. In the survival analysis, compared to the non-AKI+non-CKD group, the other 3 groups had significantly higher mortality rates (Figure). 

**Table 3:** Baseline demographic, clinical characteristics and laboratory values of the patients subgrouped according to the presence of AKI and/or CKD.

Characteristic		All patients(N = 621)	Non-AKI+non-CKD(N = 261)	AKI+non-CKD(N = 56)	Non-AKI+CKD(N = 158)	AKI+CKD(N = 146)	p
Demographic information							
Age (years), median (Q1–Q3)		60 (47–73)	49 (37–61)c d	48.5 (42–61)c d	70 (60–77)a b	69 (60–78)a b	<0.001
Sex, n/N (%)	Female	274/621 (44.1)	111/261 (42.5)	20/56 (35.7)	83/158 (52.5)	60/146 (41.1)	0.074
Smoking, n/N (%	Not smoking	391/455 (85.9)	161/187 (86.1)	28/40 (70)c	110/119 (92.4)b	92/109 (84.4)	0.005
	Smoking	64/455 (14.1)	26/187 (13.9)	12/40 (30)c	9/119 (7.6)b	17/109 (15.6)	
Coexisting disorder, n/N (%)	Diabetes mellitus	172/614 (28.0)	31/259 (12)c d	11/54 (20.4)c d	65/158 (41.1)a b	65/143 (45.5)a b	<0.001
	Hypertension	332/615 (54.0)	66/258 (25.6)c d	17/55 (30.9)c d	120/157 (76.4)a b d	129/145 (89)a b c	<0.001
	Ischemic heart disease	139/598 (23.2)	19/255 (7.5)c d	5/51 (9.8)c d	47/154 (30.5)a b d	68/138 (49.3)a b c	<0.001
	Heart failure	78/603 (12.9)	9/258 (3.5)c d	5/53 (9.4)c d	24/153 (15.7)a b d	40/139 (28.8)a b c	<0.001
	Ischemic heart disease/heart failure	161/602 (26.7)	22/256 (8.6)c d	9/53 (17)d	51/153 (33.3)a d	79/140 (56.4)a b c	<0.001
	COPD	83/606 (13.7)	25/260 (9.6)	7/51 (13.7)	26/155 (16.8)	25/140 (17.9)	0.073
	Cancer	32/602 (5.3)	9/258 (3.5)	4/51 (7.8)	11/155 (7.1)	8/138 (5.8)	0.334
	Chronic liver disease	5/609 (0.8)	3/259 (1.2)	0/53 (0)	0/156 (0)	2/141 (1.4)	0.414
Medications, n/N (%)	ACE inhibitors	103/572 (18.0)	25/253 (9.9)c d	3/53 (5.7)c d	37/143 (25.9)a b	38/123 (30.9)a b	<0.001
	ARBs	106/569 (18.6)	16/252 (6.3)c d	5/54 (9.3)c d	43/139 (30.9)a b	42/124 (33.9)a b	<0.001
	Calcium channel blockers	186/580 (32.1)	32/253 (12.6)c d	10/55 (18.2)c d	67/146 (45.9)a b	77/126 (61.1)a b	<0.001
	Beta-blockers	153/578 (26.5)	23/254 (9.1)c d	7/55 (12.7)c d	55/140 (39.3)a b	68/129 (52.7)a b	<0.001
	Other antihypertensives	85/560 (15.2)	16/254 (6.3)c d	3/52 (5.8)c d	37/137 (27)a b	29/117 (24.8)a b	<0.001
	Insulin	83/571 (14.5)	8/254 (3.1)c d	4/53 (7.5)d	33/143 (23.1)a	38/121 (31.4)a b	<0.001
	Oral antidiabetics	98/580 (16.9)	24/256 (9.4)c d	7/55 (12.7)	39/145 (26.9)a	28/124 (22.6)a	<0.001
	Statins	86/562 (15.3)	12/253 (4.7)c d	5/54 (9.3)d	29/137 (21.2)a	40/118 (33.9)a b	<0.001
	Antiaggregant or anticoagulants	188/579 (32.5)	37/253 (14.6)c d	5/51 (9.8)c d	71/145 (49)a b	75/130 (57.7)a b	<0.001
Primary kidney disease, n/N (%)	Diabetic nephropathy	83/216 (38.2)	-	-	41/107 (38.3)	42/109 (38.5)	-
	Hypertensive nephrosclerosis	109/216 (50.7)	-	-	56/107 (52.3)	53/109 (48.6)	
	ADPKD	2/216 (0.9)	-	-	2/107 (1.9)	0/109 (0)	
	Primary glomerular disease	10/216 (4.6)	-	-	2/107 (1.9)	8/109 (7.3)	
	Urologic diseases	8/216 (3.7)	-	-	3/107 (2.8)	5/109 (4.6)	
	Other	4/216 (1.8)	-	-	3/107 (2.8)	1/109 (0.9)	
Laboratory findings, Median (Q1–Q3)	Creatinine (µmol/L)	90.17 (70.72–141.4)	70.72 (53.04 – 79.56)c d	70.72 (61.88 – 83.54)c d	132.60 (106.08 – 194.48)a b d	158.68 (123.76–232.49)a b c	<0.001
	Albumin (g/L)	37.0 (33.0–40.0)	39.0 (36.0–42.0)c d	39.0 (35.0–42.0)c d	36.0 (32.5–39.0)a b d	33.0 (28.0–37.1)a b c	<0.001
	Ferritin (µg/L)	230.0 (102.0–496.0)	166.2 (84.0–355.0) d	185.5 (87.0–444.0)d	231.0 (98.0–508.5)d	374.0 (186.0–631.3)a b c	<0.001
	Haemoglobin (g/L)	123.2 ± 121.1	131.5 ± 17.9c d	128.4 ± 19.0c d	118.3 ± 21.1a b d	111.7 ± 20.2a b c	<0.001
	Lymphocyte count (×109/L)	1.200 (0.750–1.770)	1.3 (0.900–1.900)c d	1.295 (0.780–1.700)	1.100 (0.680–1.580)	0.990 (0.600–1.590)	<0.001
	Platelet count (x109/L)	223 (176–276)	215 (176–262)	232 (184–277)	228 (177–275)	231 (173–315)	0.298
C–reactive protein, n/N (%), (× upper limit)	<10	411/621 (66.2)	211/261 (80.8)c d	39/56 (69.6) d	96/158 (60.8)a d	65/146 (44.5)a b c	<0.001
	≥10	210/621 (33.8)	50/261 (19.2)c d	17/56 (30.4)d	62/158 (39.2)a d	81/146 (55.5)a b c	
Time between first symptom and diagnosis (days), Median (Q1–Q3)		3 (2–5)	3 (2–5)	3 (2–5)	3 (2–5)	4 (3–5)	0.198
Clinical presentation, n / N (%)	Mild–Moderate disease	435/619 (70.3)	230/260 (88.5)b c d	40/55 (72.7)a d	107/158 (67.7)a d	58/146 (39.7)a b c	<0.001
	Severe–Critical disease	184/619 (29.7)	30/260 (11.5)b c d	15/55 (27.3)a d	51/158 (32.3)a d	88/146 (60.3)a b c	
Radiological confirmation n/N (%)		530/621 (85.3)	208/261 (79.7)c	45/56 (80.4)	146/158 (92.4)a	131/146 (89.7)	0.001
PCR confirmation, n/N (%)			169/261 (64.8)c	43/56 (76.8)c	76/158 (48.1)a b c	94/146 (64.4)c	<0.001
Drug treatments, n/N (%)	Oseltamivir	423/568 (74.5)	174/243 (71.6)	44/53 (83)	106/152 (69.7)	99/120 (82.5)	0.030
	Macrolides	543/597 (91)	230/251 (91.6)	51/55 (92.7)	143/154 (92.9)	119/137 (86.9)	0.282
	Chloroquine / hydroxychloroquine	608/619 (98.2)	256/260 (98.5)	55/56 (98.2)	156/158 (98.7)	141/145 (97.2)	0.863
	Lopinavir–ritonavir	31/456 (6.8)	1/190 (0.5)c d	2/42 (4.8)	8/124 (6.5)a d	20/100 (20)a c	<0.001
	Favipiravir	201/520 (38.7)	50/206 (24.3)b d	21/47 (44.7)a	44/136 (32.4)a d	86/131 (65.6)a c	<0.001
	Glucocorticoids	35/450 (7.8)	3/191 (1.6)b d	6/43 (14)a	5/121 (4.1)a d	21/95 (22.1)a c	<0.001
	Tocilizumab	12/448 (2.7)	1/191 (0.5)b	5/43 (11.6)a c	2/122 (1.6)b	4/92 (4.3)	0.001
	Convalescent plasma	1/442 (0.2)	0/191 (0)	1/41 (2.4)	0/120 (0)	0/90 (0)	–
	Canakinumab/anakinra	1/444 (0.2)	0/191 (0)	0/42 (0)	0/120 (0)	1/91 (1.1)	–
Laboratory tests during hospitalization, n / N (%)	Leukopenia (<4.00×10 9/ L)	100/615 (16.3)	40/258 (15.5)	11/54 (20.4)	25/157 (15.9)	24/146 (16.4)	0.851
	Lymphopenia (<1.50×109/ L)	329/613 (53.7)	105/256 (41)c d	30/55 (54.5)d	86/156 (55.1)a d	108/146 (74)a b c	<0.001
	Anaemia (haemoglobin<100 g/L)	166/615 (27.0)	17/258 (6.6)b c d	12/56 (21.4)a d	50/156 (32.1)a d	87/145 (60)a b c	<0.001
	Thrombocytopenia (<150.00x109/L)	96/614 (15.6)	23/257 (8.9)d	10/55 (18.2)	22/157 (14)d	41/145 (28.3)a c	<0.001
	LDH (>2×upper limit of normal)	158/603 (26.2)	23/253 (9.1)b c d	22/53 (41.5)a	39/155 (25.2)a d	74/142 (52.1)a c	<0.001
	AST (>2×upper limit of normal)	129/612 (21.1)	26/255 (10.2)b d	15/56 (26.8)a	29/157 (18.5)d	59/144 (41)a c	<0.001
Highest value of CRP level during follow-up, n / N (%)	Normal	96/619 (15.5)	67/261 (25.7)c d	15/56 (26.8)c d	11/157 (7)a b	3/145 (2.1)a b	<0.001
	1–5	151/619 (24.4)	85/261 (32.6)c d	14/56 (25)	32/157 (20.4)a	20/145 (13.8)a	
(x of upper normal value)	5–10	85/619 (13.7)	38/261 (14.6)	2/56 (3.6)	25/157 (15.9)	20/145 (13.8)	
	10–20	120/619 (19.4)	42/261 (16.1)	11/56 (19.6)	41/157 (26.1)	26/145 (17.9)	
	> 20	167/619 (27.0)	29/261 (11.1)b c d	14/56 (25)a	48/157 (30.6)a	76/145 (52.4)a b c	
Length of stay at hospital (days), Median (Q1–Q3)		9 (6–14)	7 (5–11)b d	9 (7–14.5)a	8.5 (6–12) d	12 (9–16)a c	<0.001
ICU admission, n / N (%)		125/621 (20.1)	12/261 (4.6) b c d	11/56 (19.6) a d	28/158 (17.7) a d	74/146 (50.7) a b c	<0.001
Mechanical ventilation n / N (%)		95/124 (76.6)	3/12 (25)d	8/10 (80)	19/28 (67.9)	65/74 (87.8)a	<0.001
Outcome, n / N (%)	Dead	86/621 (13.8)	2/261 (0.8)b c d	8/56 (14.3)a d	16/158 (10.1)a d	60/146 (41.1)a b c	<0.001
	Discharged	518/621 (83.4)	256/261 (98.1)b c d	46/56 (82.1)a d	137/158 (86.7)a d	79/146 (54.1)a	
	Transfer to another center	7/621 (1.1)	2/261 (0.8)	1/56 (1.8)	2/158 (1.3)	2/146 (1.4)	
	Still in ICU	10/621 (1.6)	1/261 (0.4)	1/56 (1.8)	3/158 (1.9)	5/146 (3.4)	

**Figure:**
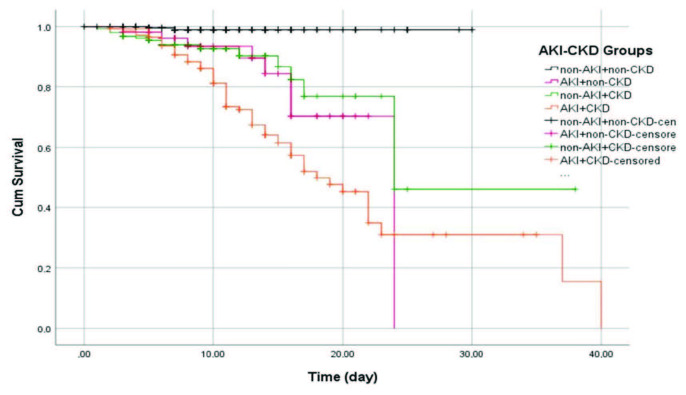
Kaplan-Meier cumulative survival analysis among AKI-CKD groups. Compared to the group without AKI + CKD, mortality rates were significantly higher in the other 3 groups.

The following parameters found to be associated with the development of AKI in univariate analysis were put into the model created to determine the independent factors associated with the development of AKI: age, sex, presence of diabetes mellitus, presence of hypertension, ischemic heart disease, or presence of heart failure, preexisting CKD, use of ACE inhibitors, beta-blocker use, clinical severity of the disease at the presentation, albumin, hemoglobin, and degree of CRP. In this model, the male sex, presence of ischemic heart disease or heart failure, severe-critical disease at presentation, hemoglobin, and preexisting CKD were found to be independent risk factors for the development of AKI during hospitalization (Table 4). The same parameters were used in the development of independent risk factors associated with mortality (instead of preexisting CKD, AKI-CKD groups were used): age, severe-critical disease at presentation, and low albumin were found as risk factors. In addition, when compared with non-AKI+non-CKD group, being in the AKI+non-CKD group (hazard ratio: 9.0, 95% CI: 1.9–44.2) or being in the AKI+CKD group (hazard ratio: 7.9, 95% CI: 1.9–33.3) showed the highest risks for mortality (Table 4).

**Table 4:** The final multivariate analyses results of the independent parameters related to the development of AKI and death during hospitalization.

	p	OR	%95 CI OR
Logistic regression analysis for AKI development			
Sex (male)	0.049	1.563	1.002–2.438
Ischemic heart disease/Heart failure	0.007	1.946	1.204–3.144
Hemoglobin (g/L)	0.025	0.878	0.783–0.983
Severe-critical disease at presentation	<0.001	2.700	1.723–4.232
Preexisting CKD	0.009	1.882	1.171–3.025
Cox regression analysis for mortality			
Age (years)	0.001	1.045	1.02–1.07
Severe-critical disease at presentation	<0.001	7.540	2.95–19.3
Serum albumin (g/L)	0.039	0.577	0.34–0.97
Non-AKI+non-CKD group (reference)	0.001		
AKI+non-CKD group	0.006	9.054	1.85–44.26
Non-AKI+CKD group	0.185	2.829	0.61–13.15
AKI+CKD group	0.005	7.878	1.86–33.34

## 4. Discussion 

This study showed that AKI was very prevalent (32.5%) among hospitalized patients with Covid-19, and it was related to worse outcomes, namely, the need for ICU admission and mechanical ventilation in ICU patients or mortality (42.1%, 86.9%, and 33.7%, respectively). Moreover, AKI incidence was more common in CKD patients as expected (48.0% vs. 17.6%), and in the case of AKI on CKD, patient mortality was much worse than non-AKI+non-CKD patients, even after multivariate analyses for adjustment of confounding factors (Table 4). On the other hand, adjusted mortality was not significantly higher in CKD patients without AKI than in non-AKI non-CKD patients (the control group of this study). These results show that CKD patients are more prone to the development of AKI. In addition, AKI, whether it develops on CKD or not, has a substantial multiplier effect on the risk of poor outcomes in hospitalized Covid-19 patients.

To the best of our knowledge, no study specifically examined HA-AKI development in CKD patients with Covid-19 patients. There are some studies that may indirectly give some supportive data to our results. In a study including 701 patients from Wuhan, it was found that the prevalence of low eGFR (<60 mL/min/1.73 m2), whether it was acute or CKD, was 13.1% on admission to the hospital [2]. AKI developed in 5.1% of the patients during hospitalization, which was significantly lower than that of our study (32.5%) [2]. Similar to our study, the incidence of AKI was almost threefold higher in patients with elevated baseline serum creatinine (11.9%) compared to patients with normal values (4.0%). They also found that in-hospital mortality was significantly higher in patients with elevated baseline serum creatinine than normal serum creatinine (33.7% vs. 13.2%). On the other hand, AKI incidence was found as 36.6% among 5449 hospitalized Covid-19 patients in a study from New York, a figure very close to our results [10]. In this study, similar to our findings, most of the AKI was stage I (46.5%). However, CKD could not be considered a comorbidity in a New York study due to diagnostic difficulties [10]. In another study from New York City, the AKI rate in the overall study patients was 33.9% (288/850), which was also very similar to our results [11]. In that study, the rate of AKI development was much higher in the ICU patients than that of the patients that did not need ICU admission (78.0% vs. 16.9%), also similar to our results (42.1% vs. 9.5%) [11]. In this study, 13.7% of the overall population had a renal disease at presentation. Although it was not clear whether these were CKD or not, the presence of renal disease at presentation was found to be the most significant independent variable in the multivariate analyses model for death [11], signifying the association between kidney disease and mortality in Covid-19 patients. In another recent preprint published study from New York, AKI occurred in 1406 (46%) of 3235 hospitalized Covid-19 patients and associated with increased mortality (41%) [12]. Moreover, there is crude data suggesting that Covid-19 may be more severe in CKD patients. In a metaanalysis, including four studies and 1389 patients, which aimed to find out the relationship between potential association between CKD and severity of Covid-19, 273 (19.7%) patients had severe disease. A significant association between CKD and severe Covid-19 was observed [OR 3.03 (95% CI: 1.09–8.47)] [13]. All these results are parallel with our findings suggesting that AKI develops more frequently in patients with underlying kidney disease, and it is related to more severe Covid-19 disease. In addition, the presence of the underlying kidney disease is related to mortality of Covid-19 patients, especially if AKI develops.

The association of AKI with mortality in Covid-19 patients might actually not be a specific occasion for Covid-19 disease. It was known that AKI develops more frequently in hospitalized patients with more severe disease, and in these patients, who develop AKI, in-hospital outcomes were worse. Moreover, the rates of AKI were reported in patients without Covid-19, and the regional distributions of these rates were largely similar to those reported in Covid-19 studies. The pooled incidence rate of AKI in adults, in the pre-Covid-19 era, was reported as 21.6% (95% CI: 19.3–24.1) in a metaanalysis, including 312 studies (n = 49,147,878) from primarily in hospital settings [14]. The incidence of AKI in these studies was reported as 21.7 to 27.5 in North America and 7.0 to 28.2 in Eastern Asia, which are similar to those reported in Covid-19 studies from those regions [14]. The mortality of patients with AKI in studies prior to Covid-19 also appears to be similar to those in studies in patients with Covid-19. Although regional differences exist, in-hospital mortality was reported as 27%–32% in the metaanalysis, which is very close to mortality in our study (33.7%) [14,15].

Moreover, the incidence of AKI is also high in other infectious diseases, which can cause pneumonia and sepsis as Covid-19. For example, in a study in which AKI complicated 18.3% of all hospitalizations, the highest in-hospital mortality rate in AKI patients was observed in the patients with sepsis (30.2%) and followed by pneumonia (27.2%) [16]. Moreover, the average age of patients included in Covid-19 studies regarding AKI was higher than most of the studies that did not include Covid-19 patients. All these data might show that the frequency and mortality of AKI in Covid-19 patients may not differ much from patients of similar severity without Covid-19 disease. Comparative studies are needed to clarify whether AKI frequency and mortality in Covid-19 patients are different from the patients without Covid-19 patients.

One of the most striking findings in our study is the relatively low mortality (0.8%) in the non-AKI+non-CKD group. This group was composed of the patients with the lowest average age, and the lowest rate of comorbidity, the best overall lab data, and the least disease severity (Table 3). However, the very low mortality rate in this group shows that kidney function may be a very important indicator of mortality during the Covid-19 outbreak. Consistent with this finding, the mortality of the non-AKI + CKD group was significantly higher than that of the non-AKI+non-CKD group (10.1% vs. 0.8%, respectively). Moreover, AKI developed in 48.0% of the patients with known preexisting-CKD, whereas it developed only in 17.6% of patients without CKD. However, contrary to mortality, these relationships with preexisting CKD and AKI development continued in multivariate analyses (Table 4), as in studies without Covid-19 patients [17]. These results show that patients with no significant change in kidney function during the Covid-19 outbreak have very good survival, even in patients with preexisting CKD.

There were some limitations of this study. As being retrospective, there was a significant lack of data in many patients. The hourly urine monitoring, fluids that were given, nephrotoxic agent use, urinary sediment, kidney imaging during hospitalization could not be gathered for the study in each patient. Moreover, there was no detailed data about the treatments in ICU in the database. However, it was challenging to collect all this data in such nationwide multicenter studies during the pandemic. We did not require the documentation of any kidney damage markers in the diagnosis of stage 3–5 CKD patients. As it is known, KDIGO also defines patients with low GFR (GFR < 60 mL/min/1.73 m2) for more than 3 months as CKD regardless of the kidney damage markers. Hence, these tests were not required to diagnose CKD in patients with stage 3–5 CKD by KDIGO. During the pandemic, we did not have the chance to perform these additional examinations (urinalysis, kidney imaging, and kidney biopsy etc.) for each patient. Therefore, this may have led to misleading results in some patients. However, all of the researchers participating in the study were nephrologists, and all evaluations, diagnoses, and records were confirmed by these researchers. This has made our data and findings more reliable and robust.

In conclusion, AKI develops more frequently in patients hospitalized due to Covid-19, and it further increases the mortality of those patients. The patients with previous CKD are more likely to develop AKI when hospitalized for Covid-19. Moreover, the outcomes of these patients are even worse. The patients without any significant change in kidney function during the Covid-19 outbreak may have better survival.

## Informed consent

This study was approved by the Local Ethics Committee of University of Health Sciences Haseki Training and Research Hospital (Protocol number 2020-41).
